# Supplementary material for: A University’s Role in Developing a Regional Network of Dementia Friendly Communities
Source: Int J Environ Res Public Health. 2025 May 1;22(5):721. doi: 10.3390/ijerph22050721 (PMC12111559; doi:10.3390/ijerph22050721)
Supplement: Supplementary file 1 [file ijerph-22-00721-s001.zip › ijerph-3471361-supplementary.pdf]

A University's Role in Developing a Regional Network of Dementia Friendly Communities: Supplementary Materials

(Standiford Reyes, Ehlman, Leahy, & Lawrence, 2025)

Table of Contents

Section 1: Supplementary Tables and Figures 3

File S1. DFC Monthly Report 3

File S2. Stakeholder and Action Team Information 17

Table S1: Rubric for Assessing Community Adherence to the Dementia Friendly Community Process 17

Table S2. Rubric for Assessing Whether Communities Exhibit DFC Principles 211

Table S3. Rubric for Assessing Alignment with DFCs Nationally: Number of Activities by National Goal 22

Table S4. Defined Acronyms 23

Section 1: Supplementary Tables and Figures

File S1. DFC Monthly Report

This figure outlines the setup details for the DFC Monthly Activity Report facilitated on Qualtrics.

**DFC Monthly Activity Report**<br>Welcome to the DFC Monthly Activity Report! We hope this report will simplify tracking across the project. Please contact (xxx) with any questions or suggestions about this form.	
**Question**	**Answer Options**
**Section 1: DFC Activities** (All will answer)	
For which community do you want to report this activity?	List Counties/ communities participating in the DFC initiative.
Which of the following activities are you reporting on? Please select all that apply.	☐ Action Team Meeting<br>☐ Community Assessment<br>☐ Community Education (e.g., Dementia Friends Training, Alzheimer's Association-led education)<br>☐ Dementia Live! (Be sure to report training of colleagues within home agencies, as applicable)<br>☐ Community Outreach (e.g, providing a presentation to a community group or staffing a booth at a community forum to share information about DFC)<br>☐ Policy Change (e.g., working with a specific organization to change its policy or procedures OR work with a community coalition to change a local ordinance)<br>☐ Support Group (i.e., Memory Cafe, Alz Assoc support group started, etc.)<br>☐ Sector Training (e.g., a training with regional transportation service, first responders or local restaurants to improve services for PLWD)<br>☐ Other
**Only answer if "Action Team Meeting" was answered to "Which of the following activities are you reporting on? Please select all that apply. "**	

Please provide the following information about the Action Team meeting. Also, please be sure to enter attendance on your DFC Action Team roster saved to Teams.	Date:<br>Meeting Location ZIP code:<br>Number of hours per meeting:<br>Number of attendees:
Was the Action Team meeting in-person or virtual?	☐ In-person<br>☐ Virtual<br>☐ Hybrid<br>☐ Conference Call
Did any new individuals join your Action Team this month?	☐ Yes (Please be sure to save contact information to your stakeholder roster saved to the DFC MS Team)<br>☐ No
If answered Yes to " Did any new individuals join your Action Team this month?"<br><br>What is the name of the new Action Team member?	
If answered Yes to " Did any new individuals join your Action Team this month?"<br><br>What sector(s) do/es the new Action Team member(s) represent? You may select more than 1 if it applies.	☐ Residential living/Memory loss supports and services<br>☐ Banks/Financial services<br>☐ Neighbors/community members<br>☐ Independent living services and supports for older adults<br>☐ Communities of faith<br>☐ Continuum of care (e.g., behavioral health, primary care, hospitals, home healthcare)<br>☐ Business/retail employers<br>☐ Local gov't emergency planning and first response (e.g., paramedics, law enforcement)<br>☐ Legal and advanced planning services<br>☐ Arts/civic engagement<br>☐ Transportation, Housing and Public Spaces (e.g., public libraries, schools, county government)<br>☐ Senior groups, organizations and advocates<br>☐ Direct care workers (e.g., an individual employed to provide hands-on support to help older adults perform everyday activities)

	☐ Caregivers (e.g., family member, friend, neighbor or others who provide unpaid assistance to older adults needing help with I/ADLs)<br>☐ Older adults living with dementia (e.g., PLWD)<br>☐ Other
If answered Yes to " Did any new individuals join your Action Team this month?"<br><br>Did any additional Action Team members join this month?	☐ Yes (Please be sure to save contact information to your stakeholder roster saved to the DFC MS Team)<br>☐ No
If answered Yes to " Did any additional Action Team members join this month?"<br><br>What is the name of the new Action Team member?	
If answered Yes to " Did any additional Action Team members join this month?<br><br>What sector(s) do/es the new Action Team member(s) represent? You may select more than 1 if it applies.	☐ Residential living/Memory loss supports and services<br>☐ Banks/Financial services<br>☐ Neighbors/community members<br>☐ Independent living services and supports for older adults<br>☐ Communities of faith<br>☐ Continuum of care (e.g., behavioral health, primary care, hospitals, home healthcare)<br>☐ Business/retail employers<br>☐ Local gov't emergency planning and first response (e.g., paramedics, law enforcement)<br>☐ Legal and advanced planning services<br>☐ Arts/civic engagement<br>☐ Transportation, Housing and Public Spaces (e.g., public libraries, schools, county government)<br>☐ Senior groups, organizations and advocates<br>☐ Direct care workers (e.g., an individual employed to provide hands-on support to help older adults perform everyday activities)

	☐ Caregivers (e.g., family member, friend, neighbor or others who provide unpaid assistance to older adults needing help with I/ADLs)<br>☐ Older adults living with dementia (e.g., PLWD)<br>☐ Other
**Only answer if "Community Assessment" was answered to "Which of the following activities are you reporting on? Please select all that apply. "**	
Which assessment activity is the DFC working on? (You may select more than one option)	☐ Dementia Friendly Community Stakeholder Assessment (from DFA)<br>☐ Basic Knowledge of Alzheimer's Disease (BKAD) assessment<br>☐ Review of data from local agency<br>☐ Other
Only answer if "Dementia Friendly Community Stakeholder Assessment (from DFA) " was answered to "Which assessment activity is the DFC working on? (You may select more than one option)"<br><br>What is the number of DFC Stakeholder Assessments conducted? Fill in the number for each of the sectors included in the assessment.	- Residential living/Memory loss supports and services: *(Fill in Number)*<br>- Banks/Financial services: *(Fill in Number)*<br>- Neighbors/community members: *(Fill in Number)*<br>- Independent living services and supports for older adults: *(Fill in Number)*<br>- Communities of faith: *(Fill in Number)*<br>- Continuum of care (e.g., behavioral health, primary care, hospitals, home healthcare): *(Fill in Number)*<br>- Business/retail employers: *(Fill in Number)*<br>- Local gov't emergency planning and first response (e.g., paramedics, law enforcement): *(Fill in Number)*<br>- Legal and advanced planning services: *(Fill in Number)*<br>- Arts/civic engagement: *(Fill in Number)*<br>- Transportation, Housing and Public Spaces (e.g., public libraries, schools, county government): *(Fill in Number)*<br>- Senior groups, organizations and advocates:*(Fill in Number)*

	- Direct care workers (e.g., an individual employed to provide hands-on support to help older adults perform everyday activities): *(Fill in Number)*<br>- Caregivers (e.g., family member, friend, neighbor or others who provide unpaid assistance to older adults needing help with I/ADLs): *(Fill in Number)*<br>- Older adults living with dementia (e.g., PLWD)<br>- Other
Only answer if "Basic Knowledge of Alzheimer's Disease (BKAD) assessment" was answered to "Which assessment activity is the DFC working on? (You may select more than one option)"<br><br>What is the number of DFC Stakeholder Assessments conducted? Fill in the number for each of the sectors included in the assessment.	- Residential living/Memory loss supports and services: *(Fill in Number)*<br>- Banks/Financial services: *(Fill in Number)*<br>- Neighbors/community members: *(Fill in Number)*<br>- Independent living services and supports for older adults: *(Fill in Number)*<br>- Communities of faith: *(Fill in Number)*<br>- Continuum of care (e.g., behavioral health, primary care, hospitals, home healthcare): *(Fill in Number)*<br>- Business/retail employers: *(Fill in Number)*<br>- Local gov't emergency planning and first response (e.g., paramedics, law enforcement): *(Fill in Number)*<br>- Legal and advanced planning services: *(Fill in Number)*<br>- Arts/civic engagement: *(Fill in Number)*<br>- Transportation, Housing and Public Spaces (e.g., public libraries, schools, county government): *(Fill in Number)*<br>- Senior groups, organizations and advocates:*(Fill in Number)*<br>- Direct care workers (e.g., an individual employed to provide hands-on support to help older adults

	perform everyday activities): *(Fill in Number)*<br>- Caregivers (e.g., family member, friend, neighbor or others who provide unpaid assistance to older adults needing help with I/ADLs): *(Fill in Number)*<br>- Older adults living with dementia (e.g., PLWD)<br>- Other
Only answer if "Review of data from local agency" was answered to "Which assessment activity is the DFC working on? (You may select more than one option)"<br><br>What sector(s) did the data come from? (i.e., what is the sector of each of the local agencies that provided data?)	☐ Residential living/Memory loss supports and services<br>☐ Banks/Financial services<br>☐ Neighbors/community members<br>☐ Independent living services and supports for older adults<br>☐ Communities of faith<br>☐ Continuum of care (e.g., behavioral health, primary care, hospitals, home healthcare)<br>☐ Business/retail employers<br>☐ Local gov't emergency planning and first response (e.g., paramedics, law enforcement)<br>☐ Legal and advanced planning services<br>☐ Arts/civic engagement<br>☐ Transportation, Housing and Public Spaces (e.g., public libraries, schools, county government)<br>☐ Senior groups, organizations and advocates<br>☐ Direct care workers (e.g., an individual employed to provide hands-on support to help older adults perform everyday activities)<br>☐ Caregivers (e.g., family member, friend, neighbor or others who provide unpaid assistance to older adults needing help with I/ADLs)<br>☐ Older adults living with dementia (e.g., PLWD)<br>☐ Other

Which of the following goals apply to the assessment activity(ies)?	☐ Learning what is and is not available in the community to PLWD and caregivers<br>☐ Identifying community educational needs<br>☐ Identifying other local groups with PLWD and caregiver memberships or interests<br>☐ Identifying ways to make community businesses and/or services more dementia-friendly<br>☐ Understanding the impact of dementia on the community<br>☐ Other
What else would you like to share about the assessment work conducted in this community or what you are learning about the needs of PLWD and caregivers?	☐
**Only answer if "Community Education" was answered to "Which of the following activities are you reporting on? Please select all that apply. "**	
Please provide the following information about the community education provided.	Date:<br>Name of Training:<br>Location ZIP code:<br>Number of hours:<br>Number of attendees:
Was the activity in-person or virtual?	☐ In-person<br>☐ Virtual<br>☐ Hybrid<br>Conference Call
Who delivered the educational session? (Mark all that apply)	☐ Greater Indiana chapter of the Alzheimer's Association<br>☐ Greater Kentucky and Southwestern chapter of the Alzheimer's Association<br>☐ Generations<br>☐ SWIRCA & More<br>☐ Other
Did any of the following groups attend the community training? If so, please fill in the number of attendees.<br>Direct care workers - individuals employed to provide hands-on support to help older adults perform I/ADLs).	Primary Care Providers:<br>Direct Care Workers:<br>Caregivers:<br>PLWD:

Caregivers - family member, friend, neighbor or others who provide unpaid assistance to older adults needing help with I/ADLs).	
Was there more than 1 community education event that you are reporting on?	☐ Yes<br>☐ No
If answered Yes to " Was there more than 1 community education event that you are reporting on? "<br><br>Please provide the following information about the community education provided.	Date:<br>Name of Training:<br>Location ZIP code:<br>Number of hours:<br>Number of attendees:
If answered Yes to " Was there more than 1 community education event that you are reporting on? "<br><br>Was the activity in-person or virtual?	☐ In-person<br>☐ Virtual<br>☐ Hybrid<br>Conference Call
If answered Yes to " Was there more than 1 community education event that you are reporting on? "<br><br>Who delivered the educational session? (Mark all that apply)	☐ Greater Indiana chapter of the Alzheimer's Association<br>☐ Greater Kentucky and Southwestern chapter of the Alzheimer's Association<br>☐ Generations<br>☐ SWIRCA & More<br>☐ Other
If answered Yes to " Was there more than 1 community education event that you are reporting on? "<br><br>Did any of the following groups attend the community training? If so, please fill in the number of attendees.<br>Direct care workers - individuals employed to provide hands-on support to help older adults perform I/ADLs).<br>Caregivers - family member, friend, neighbor or others who provide unpaid assistance to older adults needing help with I/ADLs).	Primary Care Providers:<br>Direct Care Workers:<br>Caregivers:<br>PLWD:
**Only answer if "Dementia Live" was answered to "Which of the following activities are you reporting on? Please select all that apply. "**	

Please provide the following information Dementia Live! Event.	Date:<br>Location ZIP code:<br>Number of hours:<br>Number of attendees:
What sector(s) participated in the Dementia Live! training? Mark all that apply.	☐ Residential living/Memory loss supports and services<br>☐ Banks/Financial services<br>☐ Neighbors/community members<br>☐ Independent living services and supports for older adults<br>☐ Communities of faith<br>☐ Continuum of care (e.g., behavioral health, primary care, hospitals, home healthcare)<br>☐ Business/retail employers<br>☐ Local gov't emergency planning and first response (e.g., paramedics, law enforcement)<br>☐ Legal and advanced planning services<br>☐ Arts/civic engagement<br>☐ Transportation, Housing and Public Spaces (e.g., public libraries, schools, county government)<br>☐ Senior groups, organizations and advocates<br>☐ Direct care workers (e.g., an individual employed to provide hands-on support to help older adults perform everyday activities)<br>☐ Caregivers (e.g., family member, friend, neighbor or others who provide unpaid assistance to older adults needing help with I/ADLs)<br>☐ Older adults living with dementia (e.g., PLWD)<br>☐ Other
**Only answer if "Community Outreach" was answered to "Which of the following activities are you reporting on? Please select all that apply. "**	
Enter the following information about community outreach efforts.	Date:<br>Name of Community Event:<br>Location ZIP code:<br>Number of hours:<br>Total number attending event:

Was the outreach activity in-person or virtual?	☐ In-person<br>☐ Virtual<br>☐ Hybrid<br>☐ Conference Call
What number of people...	- Signed up to be on the local DFC distribution list*: (Fill in Number)*<br>- Picked up dementia education materials*: (Fill in Number)*<br>- Signed up to volunteer at a project or event*: (Fill in Number)*<br>- Asked to attend an Action Team meeting*: (Fill in Number)*<br>- Picked up information on local community resources for PLWD and/or caregivers*: (Fill in Number)*<br>- Signed up to attend an event with the Alzheimer's Association*: (Fill in Number)*<br>- Other (please activity and #)*: (Fill in Number and activity)*
**Only answer if "Support Group" was answered to "Which of the following activities are you reporting on? Please select all that apply. "**	
What is the policy that the DFC is engaged in changing?	
What is the scope of this policy?	☐ Individual business or organization<br>☐ Association or collaborating group of businesses and organizations<br>☐ One or more sectors<br>☐ Whole community (i.e., local ordinance)
What sector(s) are participating in the policy change work? Mark all that apply.	☐ Residential living/Memory loss supports and services<br>☐ Banks/Financial services<br>☐ Neighbors/community members<br>☐ Independent living services and supports for older adults

	☐ Communities of faith<br>☐ Continuum of care (e.g., behavioral health, primary care, hospitals, home healthcare)<br>☐ Business/retail employers<br>☐ Local gov't emergency planning and first response (e.g., paramedics, law enforcement)<br>☐ Legal and advanced planning services<br>☐ Arts/civic engagement<br>☐ Transportation, Housing and Public Spaces (e.g., public libraries, schools, county government)<br>☐ Senior groups, organizations and advocates<br>☐ Direct care workers (e.g., an individual employed to provide hands-on support to help older adults perform everyday activities)<br>☐ Caregivers (e.g., family member, friend, neighbor or others who provide unpaid assistance to older adults needing help with I/ADLs)<br>☐ Older adults living with dementia (e.g., PLWD)<br>☐ Other
Which of the following best represents the focus of the DFC's policy change work at this time? Mark all that apply.	☐ Research to better understand the problem<br>☐ Research to identify alternatives<br>☐ Educating policy makers to discuss the problem<br>☐ Organizing impacted individuals to engage in policy change work<br>☐ Working with a champion policy maker to draft the policy change<br>☐ Obtaining a public hearing with institutional decision makers<br>☐ Obtaining a vote on new policy<br>☐ Policy has been changed
**Only answer if "Support Group" was answered to "Which of the following activities are you reporting on? Please select all that apply. "**	

Mark the response(s) that best represent the main areas of work with support group at this time.	☐ Identifying/recruiting a support group leader<br>☐ Identifying a host organization<br>☐ Identifying where/when to hold the meetings<br>☐ Training and supporting the leader<br>☐ Engaging other community based organizations for referrals<br>☐ Providing education and resources to group members<br>☐ Engaging members in sustaining the group<br>☐ Other
Do you have a support group meeting of participants to report on?	☐ Yes<br>☐ No
If answered Yes to " Do you have a support group meeting of participants to report on?"<br><br>Enter the following information a support group held.	Date:<br>Location ZIP code:<br>Number of hours:<br>Number of new attendees:<br>Total number of attendees:
If answered Yes to " Do you have a support group meeting of participants to report on?"<br><br>Who is the support group for? (you will have the opportunity to add information on more support groups if you have more than one)	☐ PLWD<br>☐ Caregivers (e.g., family member, friend, neighbor or others who provide unpaid assistance to older adults needing help with I/ADLs)<br>☐ Direct care staff (e.g., individuals employed to provide hands-on support to help older adults perform I/ADLs)
If answered Yes to " Do you have a support group meeting of participants to report on?"<br><br>Was the support group activity in-person or virtual?	☐ In-person<br>☐ Virtual<br>☐ Hybrid
If answered Yes to " Do you have a support group meeting of participants to report on?"<br><br>Was there another support group offered?	☐ Yes<br>☐ No
If answered Yes to "Was there another support group offered?"<br><br>Enter the following information a support group held.	Date:<br>Location ZIP code:<br>Number of hours:<br>Number of new attendees:<br>Total number of attendees:

If answered Yes to "Was there another support group offered?"<br><br>Who is the support group for? (you will have the opportunity to add information on more support groups if you have more than one)	☐ PLWD<br>☐ Caregivers (e.g., family member, friend, neighbor or others who provide unpaid assistance to older adults needing help with I/ADLs)<br>☐ Direct care staff (e.g., individuals employed to provide hands-on support to help older adults perform I/ADLs)
If answered Yes to "Was there another support group offered?"<br><br>Was the support group activity in-person or virtual?	☐ In-person<br>☐ Virtual<br>Hybrid
If answered Yes to "Was there another support group offered?"<br><br>Was there another support group offered?	☐ Yes<br>☐ No
If answered Yes to "Was there another support group offered?"<br><br>Enter the following information a support group held.	Date:<br>Location ZIP code:<br>Number of hours:<br>Number of new attendees:<br>Total number of attendees:
If answered Yes to "Was there another support group offered?"<br><br>Who is the support group for? (you will have the opportunity to add information on more support groups if you have more than one)	☐ PLWD<br>☐ Caregivers (e.g., family member, friend, neighbor or others who provide unpaid assistance to older adults needing help with I/ADLs)<br>☐ Direct care staff (e.g., individuals employed to provide hands-on support to help older adults perform I/ADLs)
If answered Yes to "Was there another support group offered?"<br><br>Was the support group activity in-person or virtual?	☐ In-person<br>☐ Virtual<br>Hybrid
**Only answer if "Sector Training" was answered to "Which of the following activities are you reporting on? Please select all that apply. "**	
Enter the following information for the training.	Date:<br>Location ZIP code:<br>Number of hours:<br>Total number of attendees:

Was the training activity in-person or virtual?	☐ In-person<br>☐ Virtual<br>☐ Hybrid
What sector(s) are participating in the policy change work? Mark all that apply.	☐ Residential living/Memory loss supports and services<br>☐ Banks/Financial services<br>☐ Neighbors/community members<br>☐ Independent living services and supports for older adults<br>☐ Communities of faith<br>☐ Continuum of care (e.g., behavioral health, primary care, hospitals, home healthcare)<br>☐ Business/retail employers<br>☐ Local gov't emergency planning and first response (e.g., paramedics, law enforcement)<br>☐ Legal and advanced planning services<br>☐ Arts/civic engagement<br>☐ Transportation, Housing and Public Spaces (e.g., public libraries, schools, county government)<br>☐ Senior groups, organizations and advocates<br>☐ Direct care workers (e.g., an individual employed to provide hands-on support to help older adults perform everyday activities)<br>☐ Caregivers (e.g., family member, friend, neighbor or others who provide unpaid assistance to older adults needing help with I/ADLs)<br>☐ Older adults living with dementia (e.g., PLWD)<br>☐ Other
What kind of support did the DFC provide to the sector?	☐ Facilitation of the training<br>☐ Community resource information<br>☐ Education/training on dementia<br>☐ Hosted and/or publicized the meetings

	☐ Supported one or more agencies to address PLWD and/or caregivers in business or strategic plans<br>☐ Other
**Only answer if "Other" was answered to "Which of the following activities are you reporting on? Please select all that apply. "**	
Enter the following information.	Date:<br>Name of Community Event:<br>Location ZIP code:<br>Number of hours:<br>Total number of attendees:
Did any of the following groups attend the community training? If so, please fill in the number of attendees.<br>Direct care workers - individuals employed to provide hands-on support to help older adults perform I/ADLs).<br>Caregivers - family member, friend, neighbor or others who provide unpaid assistance to older adults needing help with I/ADLs).	Primary Care Providers:<br>Direct Care Workers:<br>Caregivers:<br>PLWD:
Was the activity in-person or virtual?	☐ In-person<br>☐ Virtual<br>☐ Hybrid<br>☐ Conference Call
**Conclusion (All will answer)**	
What were 1-2 stand-out results from this reporting period?	
What, if any, challenges or lessons learned can you share from this reporting period?	

File S2. Stakeholder and Action Team Information

This Excel sheet offers a detailed guide for recording information about the stakeholders and the Action Team Members. (Please see additional formatted excel sheet.)

Table S1: Rubric for Assessing Community Adherence to the Dementia Friendly Community Process

Table S1 presents a rubric for assessing community adherence to the Dementia Friendly Community (DFC) process, focusing on key activities across various phases of implementation.

Phase	Key Activities	Com. 1A	Com. 1B	Com. 2A	Com. 2B	Com. 3A	Com. 3B	Com. 4A	Com. 4B
**Convene**	1. Convene key community leaders, citizens and PLWD to determine community readiness to embark, evaluate and sustain DFC	1	1	.5	.5	.5	.5	.5	.5
	2. Build the community case for becoming more dementia friendly	.5	.5	.5	.5.	.5	.5	.5	.5
	3. Build an Action Team consisting of key collaborator s, PLWD, family and care partners, and community members representing different sectors	1	1	1	.5	1	1	1	1
	4. Engage the community more broadly by kicking off the effort in a community meeting or event	1	1	.5	.5	.5	.5	1	1

Rating scale: 0 = no evidence of activity; .5 = evidence of partial implementation; 1 = evidence of significant implementation activity. This scale differs from subsequent scales to weight Phase One equally to items from subsequent phases.
Com. is used to abbreviate community.

Phase	Key Activities	Com. 1A	Com. 1B	Com. 2A	Com. 2B	Com. 3A	Com. 3B	Com. 4A	Com. 4B
**Engage**	Engage key or broad community membership in dialogue to learn community strengths, gaps and priorities for action	2	2	2	1	2	2	1	1
	Identify ways the community can build on its assets and fill gaps to provide additional support	1	1	1	1	1	1	0	0
Phase	Key Activities	Com. 1A	Com. 1B	Com. 2A	Com. 2B	Com. 3A	Com. 3B	Com. 4A	Com. 4B
**Analyze**	Compile and interpret data gathered during phase 2	1	1	1	1	0	0	0	0
	Use compiled data to draw conclusions and select priority goals for the community to consider undertaking	0	0	0	0	0	0	0	0

Phase	Key Activities	Com. 1A	Com. 1B	Com. 2A	Com. 2B	Com. 3A	Com. 3B	Com. 4A	Com. 4B
**Act**	Share the results and involve the community	0	0	0	0	0	0	0	0
	Develop an action plan that prioritizes opportunities and community goals and seek necessary funding to support various aspects of the work	1	1	1	1	1	1	1	1

Rating scale: 0 = no evidence of activity; 1 = evidence of partial implementation; 2 = evidence of significant implementation activity.

Com. stands for Community.

Table S2. Rubric for Assessing Whether Communities Exhibit DFC Principles

Table S2 outlines a rubric for evaluating whether communities exhibit the core principles of Dementia Friendly Communities, such as involving people living with dementia and maintaining a collaborative team.

DFC Principles	Com. 1A	Com. 1B	Com. 2A	Com. 2B	Com. 3A	Com. 3B	Com. 4A	Com. 4B
Including and involving people living with dementia in the community effort	2	2	1	1	1	1	1	1
Establishing and maintaining a team that works collaboratively to create change	2	2	2	1	2	2	2	2

Goals were specified in *Dementia-Friendly Community Evaluation Guide*, downloaded as a part of the Community Toolkit Resources available from Dementia Friendly America's website.

Rating scale: 0 = no evidence of principle; 1 = evidence of partial or episodic attention to principle; 2 = evidence of ongoing attention to principle.

Com. stands for Community.

Table S3. Rubric for Assessing Alignment with DFCs Nationally: Number of Activities by National Goal

Table S3 provides a rubric for assessing the alignment of community activities with national DFC goals, categorizing efforts to raise awareness, improve environments, and support people living with dementia.

DFC Goals	Com. 1A	Com. 1B	Com. 2A	Com. 2B	Com. 3A	Com. 3B	Com. 4A	Com. 4B
1. Increase awareness and understanding of dementia and of people living with dementia (PLWD)	2	3	1	2	1	2	1	1
2. Increase awareness and understanding of brain health and risk reduction		1		4	1			
3. Collaborate with public, private, nonprofit and health care sectors to better serve PLWD and care partners								
4. Address the changing needs of people with dementia and care partners	5	2	5	8	2	1		
5. Create a supportive		2	2		1			

social, cultural and business environment that is inclusive of those living with dementia								
6. Improve the physical environment in public places and systems (e.g., parks) to be dementia-friendly					1			

Goals were specified in *Dementia-Friendly Community Evaluation Guide*, downloaded as a part of the Community Toolkit Resources available from Dementia Friendly America's website. Analysts used the example activities provided in the guide to determine goal alignment of each regional DFC community events.
Com. stands for Community.

Table S4. Defined Acronyms

This table defines various acronyms used throughout the article such as DFA, DFC, and PLWD.

**AAA**	Area Agency on Aging
**AARP**	American Association of Retired Persons
**BKAD**	Basic Knowledge of Alzheimer's Disease
**DFA**	Dementia Friendly America
**DFC**	Dementia Friendly Communities
**GWEP**	Geriatric Workforce Enhancement Program
**MPA**	Multisector Plans on Aging
**MUA**	Medically Underserved Area
**PLWD**	People living with dementia
**WHO**	World Health Organization
